# Unfulfilled expectations of equal, respectful, or different treatment: Complaints of perceived ethnic, religious and gender discrimination in Swedish healthcare

**DOI:** 10.1177/14034948251393867

**Published:** 2025-11-24

**Authors:** Lise Eriksson, Aje Carlbom, Birgitta Essén

**Affiliations:** 1 Department of Women’s and Children’s Health, Uppsala University, Sweden; 2 Department of Social Sciences, Åbo Akademi University, Finland; 3 Department of Social Work, Malmö University, Sweden

**Keywords:** Ethnic discrimination, gender discrimination, perceived discrimination, religious discrimination, migrant healthcare, Sweden, Equality Ombudsman

## Abstract

**Aim:**

To analyse the occurrence and significance of complaints to the Equality Ombudsman (Diskrimineringsombudsmannen (DO)) of ethnic, religious and gender discrimination in Swedish healthcare in relation to discrimination as a social phenomenon.

**Methods:**

Through descriptive statistics and content analysis, we analysed complaints of discrimination on the grounds of ethnicity, religion and gender from 2012 to 2021 (*N*=431), and DO’s case supervision decisions (*n*=23).

**Results:**

DO has legally reviewed or initiated a supervisory process for only 23 (5%) of 431 cases and filed two lawsuits in the sample. Two-thirds of complainants were patients (*n*=284, 66%), one-fourth relatives (*n*=109, 25%), two healthcare professionals (1%) and 36 unspecified (8%). The most frequently reported healthcare sectors were primary healthcare centres (*n*=133, 31%), emergency care (*n*=50, 12%), women’s healthcare (*n*=47, 11%) and psychiatry/psychotherapy (*n*=40, 9%). Perceived discrimination concerned unfulfilled expectations of equal treatment (*n*=158, 37%), respectful treatment (*n*=151, 35%), different diagnosis or treatment (*n*=88, 20%), faster processing (*n*=15, 4%) and other (*n*=19, 4%).

**Conclusions:**

**Complaints of ethnic, religious and gender discrimination in Swedish healthcare often report subtle discrimination or unmet expectations, and the reported discrimination is rarely legally confirmed. The results suggest that many reported cases are likely based on misunderstandings and communication problems. The unfulfilled expectations were often related to miscommunication, distrust or perceptions of being neglected. Healthcare providers should recognise these perceptions earlier and clarify any misunderstandings. The findings point to a need for further analysis of perceived discrimination in healthcare, and that more cases should be investigated.**

## Introduction

Equal care, accessible care, patient participation and evidence-based care are the guiding principles of healthcare, according to the Swedish Patient Act (2014:821). There is reason to examine patients’ and their relatives’ reports of discrimination in healthcare, which goes against these principles. Discrimination means excluding, disadvantaging or differentiating between individuals, often across multiple social group affiliations, such as ethnicity, religion and gender. Disadvantaged individuals among minorities might face discrimination across various aspects of their identity, which can lead to cumulative stress [1–3].

This article focuses on three aspects of social diversity – ethnicity, religion and beliefs, and gender identity – owing to the importance of addressing discrimination in healthcare through intersectional analysis. Our previous research has identified tensions between policies on gender equality and cultural and religious diversity within Swedish healthcare, which motivates us to investigate the need for policy development for more equitable and respectful care in relation to ethnicity, religion and gender [4,5]. Research suggests that minorities report discrimination in healthcare to a greater degree than their majority peers [6]. Inequalities in healthcare access and discrimination may affect members of vulnerable groups, such as migrants, religious minorities, gender minorities and persons with complex medical needs [7]. Perceived discrimination in healthcare has a double negative effect by preventing equal access to healthcare and by diminishing trust in healthcare professionals and the healthcare system, thus contributing to health inequalities and worse health outcomes for disadvantaged groups [8]. Research suggests that gender inequalities in healthcare include unequal healthcare access, differential treatment of male and female patients, and non-binary persons being side-stepped due to healthcare being systemically gendered [2,7]. Many studies have shown that migrants and persons of ethnic minority backgrounds might encounter inequalities in healthcare contributing to delays or barriers in obtaining timely and accessible care [9–12]. However, little is known about the extent of discrimination by healthcare providers in a Scandinavian setting or the best methods for analysing it. Hence, this study fills a knowledge gap by conceptualising healthcare discrimination complaints and analysing their occurrence in Swedish healthcare through a mixed methods approach of descriptive statistics and content analysis.

The Swedish population is ethnically and culturally diverse, as 27% are foreign-born or born in Sweden with two foreign-born parents [13]. Sweden has adopted multiculturalism as an integration policy to promote equality and to prevent discrimination and racism [14–16]. Furthermore, the policy approach of gender mainstreaming is a central strategy to combat gender discrimination in Sweden by integrating a gender perspective in all decision-making processes rather than treating gender equality as a separate issue [4].

Persons who have experienced unfavourable treatment in Swedish public institutions may complain to the Equality Ombudsman (in Swedish, Diskrimineringsombudsmannen (DO)). To be considered as discrimination in terms of the Discrimination Act (2008:567), the incident must have a connection to at least one of the seven legal grounds of discrimination: sex, transgender identity or expression, ethnicity, religion or other belief, disability, sexual orientation, and age. Furthermore, the Discrimination Act distinguishes the following forms of discrimination: direct or indirect discrimination, inadequate accessibility, harassment, sexual harassment, and instructions to discriminate.

Healthcare was the fourth largest societal sector of reported discrimination in Sweden in 2021, encompassing 13% of all complaints received by DO. Disability was most common, and ethnicity was second most common among complaints during 2015–2020. During the COVID-19 pandemic in 2021, age discrimination was most frequently reported, followed by disability, ethnicity, gender and religion [17].

The term ‘perceived discrimination’ is relevant to understand subjective experiences of discrimination. Discrimination can take place on an institutional level through policies or practices, or on an interpersonal level in interactions between individuals [18]. While outright discrimination might have been reduced through legislation, more subtle or discreet forms of discrimination can still be experienced despite sometimes falling outside the legal definitions of prohibited grounds of discrimination. Our study contributes to examining these limits of the legal definitions of discrimination. Research also indicates that perceived discrimination has negative effects on mental health [1,3].

A study of patient-reported discrimination in the US healthcare system found that perceived discrimination appeared more common than previously recognised, although the prevalence was lower compared with other areas of society, for example, housing [19]. Perceived discrimination among patients from healthcare providers can result in non-adherence to physicians’ recommendations [20], patient disengagement from healthcare [21] and perceived lesser quality of care [22,23]. Previous research has also found that some migrants’ cultural or religious views on contraception, abortion and reproductive health might conflict with healthcare providers’ approaches to gender equality in reproductive healthcare [4,5]. Socioeconomic status, suboptimal healthcare and limited healthcare access due to miscommunication, language difficulties, poor communication between women and healthcare professionals, and limited knowledge about the Swedish healthcare system have been reported as explanations for migrants’ poor sexual and reproductive health [9,10,24,25].

The aim is to analyse the occurrence and significance of complaints to DO of ethnic, religious and gender discrimination in Swedish healthcare in relation to discrimination as a social phenomenon. To our knowledge, no studies beyond DO’s statistical reports have been conducted in Swedish healthcare, and the significance of the frequency of complaints requires interpretation [17]. We analyse the following research questions: (1) What characterises formal complaints of ethnic, religious and gender discrimination? (2) In which healthcare sectors has discrimination been reported? (3) To what extent and on what grounds have the complaints been investigated by DO?

## Methods

### Study design and sample

We used a mixed method of descriptive statistics and content analysis of a sample including complaints based on ethnicity, religion or beliefs, and gender from 2012 to 2021, as a decade provides a substantial timeframe to analyse patterns. This selection of grounds of discrimination is relevant for understanding systemic inequities and intersections in complex forms of discrimination, and we analyse these grounds of discrimination both separately and in combination.

### Variables

We answer the first research question through content analysis and descriptive statistics of complainants and complaints. The second question’s variable healthcare sectors refers to our categorisation of reported healthcare institutions or clinics from a list we received from DO and by reading the complaints. We answer the third research question through a flow chart of filed complaints, DO’s investigation decisions, and court cases, and through content analysis of investigated cases.

### Research data

We made an official request for complaints that were registered by DO on the selected grounds of discrimination. Most complaints include a form and a written account of the perceived discrimination. The authority enclosed lists with information about grounds of discrimination, the reported healthcare institutions, and dates of registration. We did not request appendices to complaints, which might include correspondence between the complainant and the notified party, images, extracts from patient records, court documents, or audio files. The data collection resulted in a total sample of 576 complaints. Two researchers constructed the analytical sample of 431 complaints by reading and categorising all 576 complaints and discussing with the other project members. We excluded 31 complaints that did not concern healthcare, as well as 114 incoherent complaints, that is, texts that were difficult to understand or extensively masked by DO, which sometimes resulted in incomprehensibility. Our data also include DO’s case supervision decisions regarding 23 complaints and court documents in relation to two complaints, and personal communication with DO through email for clarifying questions. Decisions and court documents are publicly available on DO’s website.

### Data analysis

The data were analysed using descriptive statistics and content analysis [26] through a mixed methods approach [27]. We sorted complaints in a spreadsheet including variables such as gender, role (patient/relative/healthcare professional), healthcare sector, grounds of discrimination, and DO’s investigation. Through a comprehensive content analysis of incident descriptions, we conducted data condensation by abstracting and summarising complaints into categories and brief descriptions in the spreadsheet, thereby identifying recurring patterns across cases. After preliminary categorisation, themes were defined and summarised in matrices, charts and figures. Our thematisation was characterised by defining themes with as little overlap as possible, which is central to content analysis [26]. The thematisation focused on the complainants’ perspective – their perceived discrimination and expressed expectations – as this secondary material does not give a complete picture of the course of events, healthcare encounters or the perspective of healthcare professionals. The analysis and construction of themes were discussed by the interdisciplinary project group, including three social scientists and a gynaecologist and obstetrician, which broadened our interpretive frameworks in relation to medical and health system knowledge and methodological skills.

### Ethical approval

The study received ethical approval by the Swedish Ethical Review Authority (2021-05382-01). Complaints submitted to DO are public documents. DO has obtained informed written consent from all complainants, which are informed that another party may request the documents. No directly identifiable data were collected, as DO masked such personal data.

## Results

### Descriptive statistics of complaints, complainants and healthcare sectors

Out of the 431 complaints, ethnicity (278) was the most reported ground of discrimination followed by gender (156) and religion (66). Sixty complaints involved two or three of the included grounds of discrimination.

Two-thirds were patients (*n*=284, 66%), on-fourth were accompanying relatives (*n*=109, 25%), two (1%) filed complaints in their role of healthcare professionals and the remaining 36 (8%) were unspecified. One healthcare professional reported a colleague for ethnic discrimination by derogatory remarks about a patient (Complaint 2020/2005) and the other complained about religious expressions in healthcare and reported losing his job owing to his secularist conviction (Complaint 2017/719). Among the 342 who stated their gender, 188 (55%) were women, 140 (41%) men and 14 (4%) transgender or other.

The most frequently reported healthcare sectors were primary healthcare centres (*n*=133, 31%), emergency care (*n*=50, 12%), women’s healthcare (*n*=47, 11%), psychiatry/psychotherapy (*n*=40, 9%), health systems (*n*=37, 9%) and surgery (*n*=36, 8%) (see Table I). Complaints in the category health systems often concerned administrative matters, healthcare costs, healthcare access or legislative changes. We categorised separately the following frequently occurring specialist care sectors: emergency care, women’s healthcare, and psychiatry/psychotherapy. Paediatric care includes outpatient and inpatient care, and youth clinics are outpatient care, separate from primary healthcare centres.

**Table I.** Healthcare sectors reported for discrimination on the grounds of ethnicity, religion and gender in Swedish healthcare, 2012–2021.

Healthcare sector	Frequency	Percentage
Primary healthcare centres	133	31%
Emergency care	50	12%
Women’s healthcare	47	11%
Psychiatry/psychotherapy	40	9%
Health system	37	9%
Surgery	36	8%
Internal medicine	27	6%
Dental care	25	6%
Other specialist care	16	4%
Paediatric care	16	4%
Youth clinics	4	1%
**Total**	**431**	**100%**

### Reported discrimination and unfulfilled expectations on healthcare

We identified three main themes by mapping the unfulfilled expectations on healthcare expressed in the complaints: equal treatment (*n*=158, 37%), respectful treatment (*n*=151, 35%) and different diagnosis or treatment (*n*=88, 20%) (see Figure 1). Complaints concerning equal treatment were based on comparisons, requesting the same or equivalent treatment as another party. The theme respectful treatment usually applied to cases when the patient or relatives felt offended in a care encounter. The theme different diagnosis or treatment related to complaints that healthcare providers have not taken the necessary measures in relation to an illness or situation. Hence, the patient or relative questioned the received diagnosis or treatment, perceived the care as insufficient and often expressed lack of trust in professional assessments or advice. The fourth largest theme, faster processing of care matters (*n*=15, 4%), described waiting for healthcare as a protracted process, but the connection with the reported grounds of discrimination was often unclear, for example, how the waiting was related to perceived racism. Nineteen complaints (4%) involved various other themes, such as self-determination, better communication or a secular environment.

**Figure 1.**
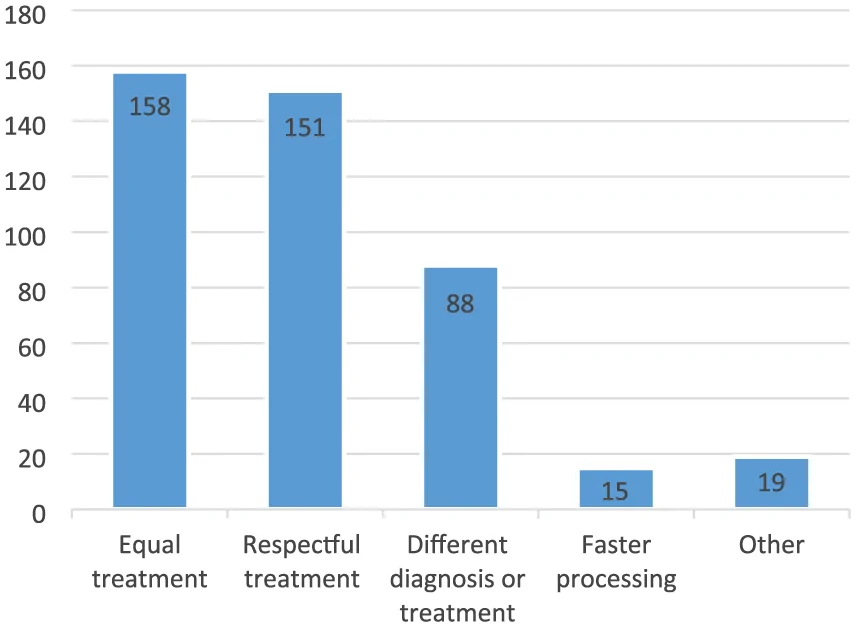
Unfulfilled expectations on healthcare expressed in complaints of perceived discrimination on the grounds of ethnicity, religion and gender in Swedish healthcare, 2012–2021.

We found an overrepresentation of ethnic minority status among the complainants, defined here as having foreign or immigrant background, dark skin colour, or minority language. Of the 278 complaints about ethnic discrimination, 232 (83%) reported ethnic discrimination in relation to ethnic minority status, while 22 (8%) reported ethnic discrimination as a Swede in comparison with positive differential treatment of migrants, and 24 were unspecified (9%). Many complaints of perceived ethnic discrimination related to healthcare providers’ questions about ethnic origin, derogatory comments and insufficient or denied care. At least one-third of the 66 religious discrimination complaints were submitted by individuals presumed to be Muslim. These complaints of religious discrimination mentioned either the patient being Muslim or wearing a hijab, niqab or veil [28]. Minority status also occurred among those reporting gender discrimination. Twelve (9%) of 134 who stated their gender had transgender or other identity and reported gender discrimination in access to gender reassignment treatments, or healthcare professionals’ condescending comments or use of undesired terms or pronouns.

Most of the complaints (*n*=393, 91%) described specific incidents in which a patient or patient’s relative experienced discrimination by healthcare professionals. We categorised 38 (9%) complaints as ‘societal’. By lacking the context of healthcare encounters, these complaints directed DO’s attention to perceived injustices in society by advocating legislative changes, increased availability of treatments, or healthcare access for specific population groups. Several societal complaints concerned perceived injustices in priorities of the COVID-19 vaccinations. Some reported perceived inequities in healthcare costs for procedures, for example, costs of prostate screening were compared with free mammography as a case of gender discrimination (Complaint 2015/1878).

### DO’s investigation of perceived discrimination

Submitted complaints are first registered by DO, including reported grounds and forms of discrimination and areas of society. An assessment by DO’s legal team of whether discrimination has occurred is made for only selected complaints [17]. DO described in personal communication the authority’s screening process of complaints as fluctuating over time, but based on a few principles:

The selection has been based either on the principle of identifying complaints that are assumed to have the most impact on discriminatory societal structures or that have contained legal issues of importance in obtaining case law. Or on the principle of identifying those which are presumed to be able to lead to redress for an affected individual. For both principles, it has been a necessary starting point that DO has assessed it likely that discrimination has taken place and that it was presumed possible to establish this out of evidentiary considerations [29].

After an initial assessment by DO’s lawyers, a limited number of cases are presented to the Head of Department, who decides whether supervision is to be initiated [29].

Figure 2 illustrates the data processing by which we constructed the analytical sample (*N*=431), the 23 cases legally reviewed by DO and two cases that proceeded to court.

**Figure 2.**
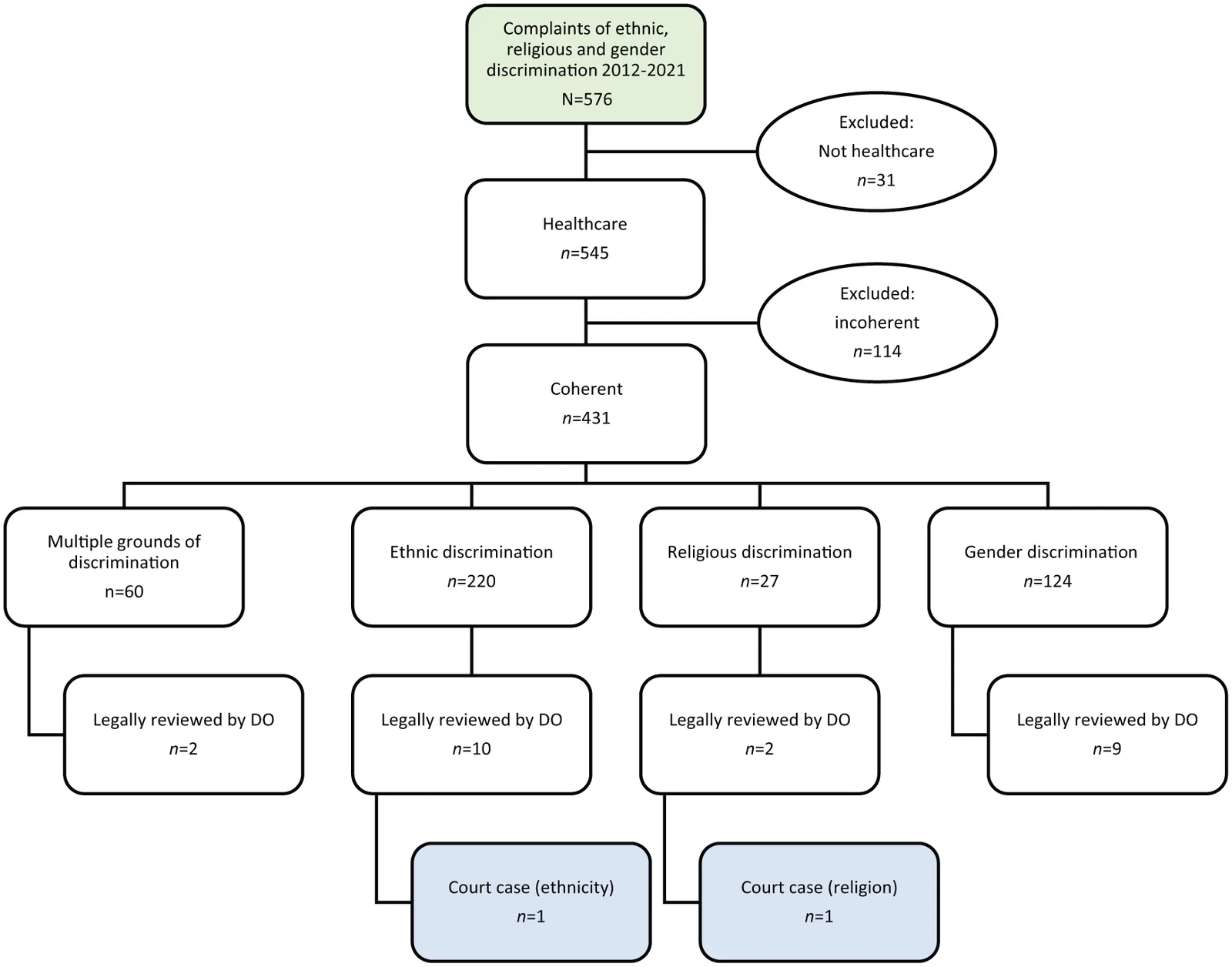
Overview of complaints of perceived discrimination on the grounds of ethnicity, religion, and gender in Swedish healthcare, 2012–2021. DO: Diskrimineringsombudsmannen [Equality Ombudsman]

The 23 legally reviewed cases are summarised in Table II with incident descriptions of perceived discrimination. Many of these complaints were detailed and argumentative, which is important for legal investigation and evidentiary requirements, and most concerned ethnic, religious or gender minorities. The cases mostly conform with thematic patterns in the larger analytical sample, including entries in patient medical records about ethnic or religious background, denied care or negative social treatment in terms of derogatory comments about skin colour, ethnicity, language or background. Two investigated complaints of religious discrimination concerned Muslim female patients. Condescending remarks about transgender identity were overrepresented among the investigated gender discrimination complaints, since five of nine concerned transgender identity (see Table II).

**Table II.** DO’s legal review of complaints of perceived discrimination on the grounds of ethnicity, religion and gender in Swedish healthcare, 2012–2021.

Reported discrimination	Unfulfilled expectations	Category	Summary of incident description in the complaint	Outcome of DO’s investigation
Ethnicity and religion	Respectful treatment	Social treatment	Jewish background was noted in patient medical records	Not discrimination
	Respectful treatment	Social treatment	Patient desired female physician at delivery and was asked to seek care in her own country if the care in Sweden does not fit the patient’s requirements	Remarks
Ethnicity	Respectful treatment	Social treatment	Harassment, condescending remarks about skin colour	Not discrimination
	Different diagnosis or treatment	Denied care	Derogatory comment about Romani ethnicity^a^; no medical treatment	No remarks
	Respectful treatment	Questions/remarks	Derogatory comment about criminal immigrant youth	Discontinued
	Equal treatment	Questions/remarks	Physician noted Romani ethnicity^a^ in patient medical records	Desired result
	Respectful treatment	Denied care	Physician commented on Swedish language skills, and denied heart surgery until patient has learned Swedish	Remarks
	Different diagnosis or treatment	Denied care	Ambulance nurse assessed cerebral haemorrhage as cultural fainting in patient medical records	Lawsuit
	Respectful treatment	Disorder attributed to ethnicity	Physician claimed that a Romani^a^ patient imagined diseases	No remarks
	Equal treatment	Denied care	Patient could not undergo surgery due to blood infection associated with adoption	No remarks
	Different diagnosis or treatment	Denied care	Physician cursed at the patient who was assessed not deserving treatment	No remarks
	Different diagnosis or treatment	Questions/remarks	Patient was questioned why not speaking Swedish and the interpreter was barely used	Not discrimination
Religion	Respectful treatment	Muslim	Physician refused to treat woman who did not want to greet with handshake	Lawsuit
	Different diagnosis or treatment	Muslim	Patient received several remarks from physician about wearing hijab	No remarks
Gender	Different diagnosis or treatment	Transgender identity	Healthcare professional used undesired designation of gender identity, and gender reassignment treatment was not started	Not discrimination
	Respectful treatment	Transgender identity	Healthcare professional used undesired designation of gender identity	Discontinued
	Different diagnosis or treatment	Social treatment	Irritated physician prohibited the patient from going to another physician	Discontinued
	Respectful treatment	Social treatment	Physician treated younger woman in a derogatory way	Not discrimination
	Equal treatment	Women’s equality	Surgery for childbirth injury was denied and seen as cosmetic surgery	No remarks
	Respectful treatment	Transgender identity	Undesired designation of gender identity	Remarks
	Equal treatment	Transgender identity	Clinic did not perform breast surgery on transgender woman	Remarks
	Respectful treatment	Transgender identity	Physician asked patient what he has between the legs and about regular sex with partner	No remarks
	Equal treatment	Gender adapted care	Patient lacked the right to free medicine, special wheelchair, and incontinence pads suitable for men	Discontinued

a The Romani people, also known as Roma, often face social exclusion, despite being one of the oldest ethnic minorities in Sweden.

DO: Diskrimineringsombudsmannen [Equality Ombudsman]

Several other complaints would have merited legal review or a supervisory process in addition to these 23 cases. For example, a woman reported ethnic discrimination by not being taken seriously during delivery and postpartum complications and was offended by being assumed to have undergone female genital mutilation or cutting and to come from a country where women have multiple children (Complaint 2020/1569). This case was also reported to the Health and Social Care Inspectorate (IVO), which considered that the complaint was justified as ethnic discrimination but not as a case of suboptimal care.

The outcome of DO’s legal review is summarised the last column of Table II. The outcome ‘Discontinued’ means that the case was closed without substantive review. ‘Remarks’ refers to DO’s assessment that discrimination had occurred, and the reported healthcare unit received remarks. ‘No remarks’ and ‘Not discrimination’ mean that DO conducted a substantive review but could not establish that the healthcare unit had violated the Discrimination Act. The outcome ‘Desired result’ refers to the investigation not providing support for violation of the Discrimination Act, but the supervision contributed to discussion about healthcare routines of how ethnicity is mentioned in patient medical records. One court case concerned the physician’s reactions to a Muslim patient’s declined greeting by handshake, but the case did not result in a conviction for religious discrimination [28]. The other court case, which resulted in conviction for ethnic discrimination, concerned a foreign-born man injured with cerebral haemorrhage during a football match. Because the man later died, the case received media attention, focusing on a remark in the ambulance records about ‘cultural fainting’:

‘very unclear. . . lying on the floor, playing unconscious. . . waking up to pain stimuli. . . whining, does not want to say so much cultural fainting. . . ??’ [30]

DO sued the Västra Götaland County Council for ethnic discrimination with reference to not providing emergency medical care within sufficient time. The County Council contested that the patient was disadvantaged and its connection to ethnicity. The County Council reported the ambulance nurse to have assessed the situation as unclear with urgent but not life-threatening symptoms, as the patient was awake on the floor when the ambulance arrived, ‘playing unconscious’. According to the judgment, downgrading of the condition to ‘cultural fainting’ was related to ethnicity, and his wife was also discriminated against by the healthcare staff not listening to her [30].

## Discussion

We found that DO has legally reviewed only 23 cases (5%) and filed two lawsuits of the analytical sample comprising 431 complaints from 2012 to 2021. However, this low proportion does not necessarily reflect the occurrence of ethnic, religious and gender discrimination in Swedish healthcare. The few legal actions by DO may be due to lack of evidence for establishing discrimination, or the authority’s lack of resources for case supervision. Since five of the 23 legally reviewed cases concerned transgender identity (see Table II), we conclude that DO has prioritised investigations of perceived discrimination against transgender identity to obtain case law or to impact societal structures [29].

The results indicate a significant gap between perceived discrimination in Swedish healthcare and what can be legally substantiated. A possible explanation is that there are differences between individuals’ everyday understanding of discrimination and the Discrimination Act’s definition of discrimination. Another interpretation is that patients and relatives can use the Discrimination Act to promote different expectations of care, which partly concern things other than discrimination and rights protected by the Discrimination Act [4,9,10]. Many complaints seem to be written in a state of emotional distress, which might reflect the stress in many healthcare situations or perceptions of being neglected. In a study analysing complaints to DO, Bursell argues that formal complaints against public institutions typically concern cases of unequal opportunities or being denied resources, and feeling neglected is prevalent in perceived healthcare discrimination [31].

Discrimination complaints categorised as societal lacked a description of a specific discriminatory incident and called for legislative changes or changes of healthcare routines. Some societal complaints might involve indirect discrimination prohibited by the Discrimination Act by involving a rule or routine that disadvantages people of a certain ethnicity, religion or gender. In theory, such complaints can contribute to DO’s work to draw attention to shortcomings in the legislation but hardly affect healthcare routines. There was an increase in complaints over time during the 10-year period, particularly since September 2020 when a web form was introduced for submitting complaints to DO. The web form facilitated complaint submission, and the vocabulary was also changed to the less formal ‘tips and complaints’, which might have contributed to an increase in societal complaints [17]. The increase was also an effect of the COVID-19 pandemic, including complaints about foreign citizens’ access to vaccination and vaccination certificates.

We present unfulfilled expectations as a conceptualisation of perceived discrimination in healthcare. The three most common unfulfilled expectations were equal treatment, respectful treatment, and different diagnosis or treatment. These were often related to challenges in miscommunication and distrust, or perceptions of being neglected in healthcare encounters. Miscommunication between providers and patients and lack of knowledge of the Swedish health system could explain why some patients who felt deprioritised in healthcare interpreted ethnic, religious or gender discrimination as the cause, despite not being clear from the complaint narrative. Case descriptions where the complainant requested respectful treatment and felt offended could sometimes be interpreted as pressured care situations, in which healthcare professionals have lost their composure. Complaints expressing expectations of different diagnosis or treatment involved disagreement in interpretations through difficulties reconciling patients’ social understanding of the subjective illness in relation to healthcare professionals’ biomedical understanding of diseases, symptoms and diagnoses [32]. For example, many of these complaints involved perceived discrimination due to denied treatment or denied sick leave. Unfulfilled expectations based on disagreements might contribute to distrust in healthcare and non-adherence to healthcare professionals’ recommendations [8,20].

In accordance with previous research [6,7], we found that minority populations reported discrimination in healthcare to a greater degree than others. This was expected owing to our inclusion criteria, particularly for perceived ethnic and religious discrimination. Bursell found that Muslims experience discrimination in Swedish healthcare largely in the form of neglect by not receiving treatment or not being listened to by healthcare providers [31]. The most frequently reported healthcare sectors, such as primary healthcare centres and emergency care, are often the first units that patients contact. In line with previous research on tensions between gender equality and migration, women’s healthcare was reported for discrimination to a relatively high degree [4,5,25]. Complaint 2020/1569, which the IVO identified as ethnic discrimination, concerns similar allegations as a previously reported court case from 2006. In that case, the municipality of Uppsala was convicted of discrimination owing to suspicion of female genital mutilation or cutting based on the ethnic background of the family. Swedish healthcare professionals are obliged to report suspicion, but if ethnic background is the only basis for suspicion and actions have been taken, then, legally, discrimination has occurred [33,34]. However, previous research on social values has found low incidence of anti-immigrant views among healthcare providers in women’s healthcare in Sweden [35]. Research from women’s healthcare has shown adverse outcome, such as perinatal and maternal mortality among migrants from low-income countries. It has been explained by socioeconomic status, but also suboptimal care due to delayed diagnosis of unusual diseases for Swedish providers, such as tuberculosis. Suboptimal care was also linked to miscommunication due to lack of interpreter use and context related misunderstandings [9,10,23,25].

The strengths of the study were unique and rich data that have rarely been analysed, and the effective integration of qualitative and quantitative data using descriptive statistics and content analysis, which increases validity. A limitation was the lack of transparency in DO’s process to identify cases that proceed to case review or supervision. Additionally, vulnerable groups such as migrants may underreport discrimination, which might result in bias in complaint reporting. We also encountered difficulties in systematically coding reported healthcare sectors and gender of complainants owing to missing data. DO provided a list of gender based on the names of some unspecified complainants, but the authority no longer maintains gender statistics inferred from names.

## Conclusions

DO has legally reviewed only 23 cases in the sample representing a decade’s discrimination complaints on the grounds of ethnicity, religion and gender. Most complaints represent patients’ and their relatives’ perceptions of neglect and their unfulfilled expectations of equal treatment, respectful treatment, or a different diagnosis or treatment. Perceived discrimination concerned behaviour, care or lack of care, and perceived injustices in society. The most frequently reported healthcare sectors were primary healthcare centres, emergency care, women’s healthcare, and psychiatry/psychotherapy, of which the first two represent patients’ first healthcare contacts in often stressful situations. To prevent misunderstandings and distrust, healthcare professionals should adopt a mindful approach to situations and medical interpretations that can be perceived as offensive. They should sometimes emphasise that a refusal of treatment or delayed care is not due to the patient’s ethnicity, religion, gender or other minority status. The findings point to a need for further analysis of perceived discrimination in healthcare, and that more cases should be investigated by DO to establish whether discrimination has occurred.

## Data Availability

The research data can be requested from DO, which makes documents available following the principle of public access to official documents and the authority’s guidelines for the disclosure of documents including personal data. The data categorisation spreadsheet is available upon reasonable request from the corresponding author.

## References

[1] Kessler RC Mickelson KD Williams DR. The prevalence, distribution, and mental health correlates of perceived discrimination in the United States. J Health Soc Behav 1999;40:208–30. 10513145

[2] Hankivsky O. Women’s health, men’s health, and gender and health: Implications of intersectionality. Soc Sci Med 2012;74:1712–20. 10.1016/j.socscimed.2011.11.029 22361090

[3] Pascoe EA Richman LS. Perceived discrimination and health: A meta-analytic review. Psychol Bull 2009;135:531. 10.1037/a0016059 PMC2747726 19586161

[4] Arousell J Carlbom A Johnsdotter S , et al. Unintended consequences of gender equality promotion in Swedish multicultural contraceptive counseling: A discourse analysis. Qual Health Res 2017;27:1518–28. 10.1177/1049732317697099 28728532

[5] Arousell J Carlbom A Johnsdotter S , et al. Does religious counselling on abortion comply with Sweden’s “women-friendly” abortion policies? A qualitative exploration among religious counsellors. Sex Cult 2019;23:1230–49.

[6] Hausmann LRM Hannon MJ Kresevic DM , et al. Impact of perceived discrimination in healthcare on patient-provider communication. Med Care 2011;49:626–33. 10.1097/MLR.0b013e318215d93c PMC3117903 21478769

[7] Orzechowski M Nowak M Bielińska K , et al. Social diversity and access to healthcare in Europe: How does European Union’s legislation prevent from discrimination in healthcare? BMC Public Health 2020;20:1399. 10.1186/s12889-020-09494-8 PMC7490891 32928175

[8] Hanssens LGM Detollenaere JDJ van Pottelberge A , et al. Perceived discrimination in primary healthcare in Europe: Evidence from the cross-sectional QUALICOPC study. Health Soc Care Community 2017;25:641–51. 10.1111/hsc.12353 27112973

[9] Essén B Bödker B Sjöberg N-O , et al. Are some perinatal deaths in immigrant groups linked to sub-optimal perinatal care services? Br J Obstet Gynaecol 2002;109:677–82. 12118647

[10] Esscher A Binder-Finnema P Bödker B , et al. Suboptimal care and maternal mortality among foreign-born women in Sweden: Maternal death audit with application of the ‘migration three delays’ model. BMC Pregnancy Childbirth 2014;14:141. 10.1186/1471-2393-14-141 PMC3998732 24725307

[11] Paradies Y Truong M Priest N. A systematic review of the extent and measurement of healthcare provider racism. J Gen Intern Med 2014;29:364–87. 10.1007/s11606-013-2583-1 PMC3912280 24002624

[12] Terhune J Dykxhoorn J Mackay E , et al. Migrant status and risk of compulsory admission at first diagnosis of psychotic disorder: A population-based cohort study in Sweden. Psychol Med 2022;52:362–71. 10.1017/S0033291720002068 PMC8842197 32578529

[13] Statistics Sweden. Population in Sweden by country/region of birth, citizenship and Swedish/foreign background, 31 December 2023, https://www.scb.Se/en/finding-statistics/statistics-by-subject-area/population/population-composition/population-statistics/pong/tables-and-graphs/foreign-born-citizenship-and-foreignswedish-background/population-in-sweden-by-countryregion-of-birth-citizenship-and-swedishforeign-background-31-december-2023/ (2023, accessed 28 June 2024).

[14] Prop. 1975:26. Regeringens proposition om riktlinjer för invandrar- och minoritetspolitiken m. m. [The Government’s bill on guidelines for immigrant and minority policy, etc.]

[15] Prop. 1997/98:16. Sverige, framtiden och mångfalden – från invandrarpolitik till integrationspolitik [Sweden, the future and diversity – from immigration policy to integration policy].

[16] Claeys A Berdai-Chaouni S Tricas-Sauras S , et al. Culturally sensitive care: Definitions, perceptions, and practices of health care professionals. J Transcult Nurs 2021;32:484–92. 10.1177/1043659620970625 33150857

[17] Equality Ombudsman. Statistik över anmälningar som inkom till Diskrimineringsombudsmannen 2015–2021 [Statistics on complaints received by the Equality Ombudsman 2015–2021], Report 2022:2. https://www.do.se/rattsfall-beslut-lagar-stodmaterial/publikationer/2022/statistik-over-anmalningar-som-inkom-till-do-2015-2021 (2022, accessed 5 June 2025).

[18] Cetrez Ö Atchulo F Garosi I , et al. Integration and determinants of psychosocial health – thematic report. Zenodo, 2021. https://doi.org/10.5281/zenodo.4647687 (2021, accessed 8 November 2025)

[19] Nong P Raj M Creary M , et al. Patient-reported experiences of discrimination in the US health care system. JAMA Netw Open 2020;3:e2029650. 10.1001/jamanetworkopen.2020.29650 PMC7739133 33320264

[20] Haywood C Lanzkron S Bediako S , et al. Perceived discrimination, patient trust, and adherence to medical recommendations among persons with sickle cell disease. J Gen Intern Med 2014;29:1657. 10.1007/s11606-014-2986-7 PMC4242876 25205621

[21] Attanasio L Kozhimannil KB. Health care engagement and follow-up after perceived discrimination in maternity care. Med Care 2017;55:830–833. 10.1097/MLR.0000000000000773 28692572

[22] Sorkin DH Ngo-Metzger Q De Alba I. Racial/ethnic discrimination in health care: Impact on perceived quality of care. J Gen Intern Med 2010;25:390. 10.1007/s11606-010-1257-5 PMC2855001 20146022

[23] Weech-Maldonado R Hall A Bryant T , et al. The relationship between perceived discrimination and patient experiences with health care. Med Care 2012;50:S62–8. 10.1097/MLR.0b013e31825fb235 PMC3726249 22895233

[24] Åkerman E Larsson EC Essén B , et al. A missed opportunity? Lack of knowledge about sexual and reproductive health services among immigrant women in Sweden. Sex Repr Healthc 2019;19:64–70. 10.1016/j.srhc.2018.12.005 30928137

[25] Almeida LM Caldas J Ayres-De-Campos D , et al. Maternal healthcare in migrants: A systematic review. Matern Child Health J 2013;17:1346–54. 10.1007/s10995-012-1149-x 23334357

[26] Miles MB Huberman AM Saldana J. Qualitative data analysis: A methods sourcebook. London: SAGE Publications, 2014.

[27] O’Cathain A Murphy E Nicholl J. Three techniques for integrating data in mixed methods studies. BMJ 2010;341: c4587. 10.1136/bmj.c4587 20851841

[28] Eriksson L Dudas V Carlbom A , et al. (2023) Perceived religious discrimination in healthcare: A qualitative study of formal complaints. In: Enstedt D Dellenborg L (eds) Culture, spirituality and religious literacy in healthcare: Nordic perspectives. Oxford/New York: Routledge, 2023, pp.90–107.

[29] Equality Ombudsman Registrar. Personal communication, email, 27 January 2022.

[30] Gothenburg District Court judgment. Case nr. T 17336-19, https://www.do.se/download/18.4719c6e31791a23945f1ac1/1622633070650/Dom-tingsratt-PRO-2019-7-tillganglig.pdf (2021, accessed 22 August 2025).

[31] Bursell M. Perceptions of discrimination against Muslims. A study of formal complaints against public institutions in Sweden. J Ethn Migr Stud 2018;47:1162–1179.

[32] Eisenberg L. Disease and illness distinctions between professional and popular ideas of sickness. Cult Med Psychiatry 1977;1:9–23. 10.1007/BF00114808 756356

[33] Johnsdotter S Essén B. Kvinnlig omskärelse/könsstympning: En kunskaps- och forskningsöversikt. Uppsala: Nationellt centrum för kvinnofrid, Uppsala University, https://kunskapsbanken.nck.uu.se/nckkb/nck/publik/fil/fulltext/705 (2011, accessed 8 November 2025).

[34] Johnsdotter S Wendel L. Prosecutions for female genital mutilation in Swedish courts: Tip of the iceberg or manifestation of epistemic injustice? Int J Law Crime Justice 2025;80:100714.

[35] Eriksson L Tibajev A Vartanova I , et al. The liberal social values of Swedish healthcare providers in women’s healthcare: Implications for clinical encounters in a diversified sexual and reproductive healthcare. Int J Public Health 2022; 67:1605000. 10.3389/ijph.2022.1605000 PMC9309245 35898801

